# Health researchers in Alberta: an exploratory comparison of defining characteristics and knowledge translation activities

**DOI:** 10.1186/1748-5908-2-1

**Published:** 2007-01-04

**Authors:** Mandi S Newton, Carole A Estabrooks, Peter Norton, Judy M Birdsell, Adeniyi J Adewale, Richard Thornley

**Affiliations:** 1 Knowledge Utilization Studies Program (KUSP), Faculty of Nursing, University of Alberta, Edmonton, Alberta, Canada; 2 Academic Head, Department of Family Medicine, University of Calgary, Calgary, Alberta, Canada; 3 Haskayne School of Business, University of Calgary, Calgary, Alberta, Canada; 4 University of Alberta, Edmonton, Alberta, Canada; 5 Alberta Heritage Foundation for Medical Research (AHFMR), Edmonton, Alberta, Canada

## Abstract

**Background:**

Canadian funding agencies are no longer content to support research that solely advances scientific knowledge, and key directives are now in place to promote research transfer to policy- and decision-makers. Therefore, it is necessary to improve our understanding of how researchers are trained and supported to facilitate knowledge translation activities. In this study, we investigated differences in health researcher characteristics and knowledge translation activities.

**Methods:**

Our sample consisted of 240 health researchers from three Alberta universities. Respondents were classified by research domain [basic (n = 72) or applied (n = 168)] and faculty [medical school (n = 128) or other health science (n = 112)]. We examined our findings using Mode I and Mode II archetypes of knowledge production, which allowed us to consider the scholarly and social contexts of knowledge production and translation.

**Results:**

Differences among health researcher professional characteristics were not statistically significant. There was a significant gender difference in the applied researcher faculty group, which was predominantly female (*p *< .05). Research domain was linked to translation activities. Applied researchers reported engaging in significantly more Mode II activities than basic researchers (*p *< .001), and scored higher than basic researchers regarding the perceived importance of translation activities (Mode I, *p *= .01; Mode II, *p *< .001). Main effects of faculty were limited to engaged dissemination (medical school < other faculties; *p *= .025) and number of publications (medical school > other faculties; *p *= .004). There was an interaction effect for research domain and faculty group for number of publications (*p *= .01), in that applied researchers in medical faculties published more than their peers in other faculty groups.

**Conclusion:**

Our findings illustrate important differences between health researchers and provide beginning insights into their professional characteristics and engagement in Mode I and Mode II activities. A future study designed to examine these dimensions in greater detail, including potential covariates across more varied institutions, would yield richer insights and enable an examination of relative influences, needs and costs of each mode of activity.

## Background

How research affects health system and patient outcomes has been a topic of increasing importance over the past decade in Canada. Two major funding agencies, the Canadian Institutes of Health Research (CIHR) and Canadian Health Services Research Foundation (CHSRF), have made strides to have research better influence policy and practice decisions by developing key directives that facilitate knowledge translation with policy- and decision-makers. While this is a much-needed focus in health care, no concomitant attention has been given to the implications of this research agenda for the health researchers themselves. We do not have a clear picture of the activities of health researchers and whether these actually align with the current funding agendas. As the group that is most active in the scholarship of discovery, it is essential to understand, enhance, and support the researcher role in order to maximize return on research investments via knowledge translation activities that specifically target the improvement of health systems and patient health outcomes.

Researchers are an inherent part of the 'knowledge production system.' This system (comprised of both knowledge creation and translation) has been examined by several academic groups and includes: research as a product [3,4], researchers and the processes used [5], knowledge translation efforts [3], and organizational context [6-8]. Gibbons and colleagues contribute to the view of the knowledge system by classifying knowledge produced within scholarly, social, and political contexts as Modes I and II [1,2]. Mode I production is reflected in traditional academic scholarship norms and values; this includes creating knowledge for creations' sake and using an academic, peer-reviewed system (e.g., publishing in high-impact, peer-reviewed journals) to regulate and safeguard research knowledge quality. Mode II knowledge production considers the influence of social and political factors. Mode II production is carried out in non-hierarchical and varied forms, and is generally situated in a specific health care context based on the needs of research end-users. As such, Mode II production typically transpires from academic to non-academic relationships (e.g., researcher and decision-maker/policy-maker collaborations) to promote research knowledge creation and transfer based on the needs of end-users in the health care system [1,2].

With funding agencies placing emphasis on Mode II production via engaged research translation activities between health researchers and decision- and policy-makers, there is potential for considerable impact on the role of researchers whose careers typically advance according to Mode I activities [9]. The use of the Gibbons et al. framework allowed us to consider the current climate of health services research for researchers; it is one that includes a funding climate that encourages Mode II production and an academic climate that encourages Mode I production [1,2]. The purpose of this paper is to report differences in characteristics and knowledge production activities across health researchers in Alberta from different research domains and faculties. Using the Mode I and Mode II archetypes as an analytical frame, we identify characteristics related to researchers' knowledge production, and consider them vis-à-vis Canada's current academic and funding conditions.

## Methods

This paper presents the Alberta-based component of a larger Canadian study on knowledge production activities (termed research transfer in the larger study). The Alberta study was supported by funds external to the national study (see acknowledgments) and involved several sub-samples: decision-makers, physicians, and researchers from medical faculties. The Alberta study also included health researchers and nurses. Data collection from researchers was consistent within both studies. The results from the larger study are published elsewhere [10-13]. The analysis reported in this paper only includes data on the researcher sub-sample, defined as researchers from faculties who are involved in health research.

### Sample

The health researcher sub-sample of the Alberta study came from three Alberta universities (Alberta, Calgary, Lethbridge), and a health researcher was defined as someone who spent at least 10% of his or her working time conducting research. Potential subjects were identified using information from the three universities. All health researchers in a clear, health-related faculty (nursing, pharmacy, rehabilitation medicine, medicine) were identified, and the names of health researchers who had been funded for health-related research were elicited. Of this potential sample, the net response rate during data collection was 60.34%, yielding a final sample of 240 researchers. The sample was further classified according to their research domain [basic (n = 72) or applied (n = 168)] and faculty [medical school (n = 128) or other health science: nursing, pharmacy, rehabilitative medicine (n = 112)] for statistical comparison.

### Data collection and study variables

Data was collected using a telephone survey aimed at capturing various aspects of the knowledge production system. The survey was a revision and extension of previous survey work carried out during the larger study [3,5]. The survey was administered between July and August 2001. Mode I and II knowledge production activities encompassed the survey's dependent variables. Mode I activity was measured by the number of peer-reviewed publications in the last five years, while Mode II activities were measured by reports of "plain dissemination" (e.g., delivering non-technical research presentations, reports) and "engaged dissemination" (e.g., involving research end-users in defining research questions, advisory committees). Independent survey variables presented in this paper include the perceived importance of knowledge translation activities as well as professional/personal demographics. ' [see Additional file 1]'.

### Data analysis

Data were analyzed using SPSS [v. 13.0]. Research characteristics and knowledge translation activity were compared using mean plots and a two (medical school vs. other faculties) × two (basic vs. applied) analysis of variance (ANOVA). Significant main and interaction effects were examined. For dichotomous variables, cross-tabulations and Chi-square tests were conducted.

## Results

There were several notable differences among health researcher professional characteristics (see Table 1). While our sample was predominantly male, applied researchers in other health science faculties were significantly more likely to be female (55%; *p *< .05). In the medical school faculty, there were comparable frequencies of academic rank; in the other faculties, applied researchers had more appointments at the assistant and associate level. Basic researchers in other health science faculties were most commonly full professors, and all basic and most applied researchers held a PhD. Medical school applied researchers had the most variation in highest degree obtained, ranging from undergraduate (3.6%) through masters level (9.6%) to PhD level (78.3%). In a comparison of years of experience after postgraduate school, basic researchers in medical faculties had the longest years of experience (M = 18.7, sd = 9.8). This group is followed by applied researchers in the same faculty (M = 16.5, sd = 7.7), closely followed by basic researchers in other health sciences faculties (M = 15.8, sd = 9.6). Applied researchers in other health sciences faculties had the least mean years of experience after postgraduate school.

**Table 1:** Sample characteristics (cross-tabulations for distribution of academic rank, work setting, education and gender by domain and faculty)

	**Medical school**		**Other faculties**	
	**Domain**		**Domain**	
**Academic Rank**	Basic	Applied	Basic	Applied
Assistant Professor	9 (20.0%)	16 (19.2%)	6 (22.2%)	22 (25.9%)
Associate. Professor	14 (31.1%)	24 (28.9%)	4 (14.8%)	32 (37.7%)
Full Professor	22 (48.9%)	39 (46.9%)	15 (55.6%)	28 (32.9%)
*Missing Cases*	0	4 (5.0%)	2 (7.4%)	3 (3.5%)
Total	45 (100%)	83 (100%)	27 (100%)	85 (100%)
				
**Work Setting**	Basic	Applied	Basic	Applied
University only	22 (48.9%)	19 (23.0%)	6 (22.2%)	22 (25.9%)
University + Hospital	18 (40.0%)	39 (46.9%)	15 (55.6%)	28 (32.9%)
*Missing Cases*	5 (11.1%)	25 (30.1%)	6 (22.2%)	35 (41.2%)
Total	45 (100%)	83 (100%)	27 (100%)	85 (100%)
				
**Education**	Basic	Applied	Basic	Applied
Bachelor	-	3 (3.6%)	-	-
Master	-	8 (9.6%)	-	8 (9.4%)
Ph.D	45 (100%)	65 (78.3%)	27 (100%)	77 (90.6%)
*Missing Cases*	0	7 (8.4%)	0	0
Total	45 (100%)	83 (100%)	27 (100%)	85 (100%)
				
**Gender**	Basic	Applied	Basic	Applied
Male	36 (80.0%)	59 (71.1%)	19 (70.4%)	38 (44.7%)
Female	9 (20.0%)	24 (28.9%)	8 (29.6%)	47 (55.3%)
*Missing Cases*	0	0	0	0
Total	45 (100%)	83 (100%)	27 (100%)	85 (100%)
				
**Years of Postgraduate Experience**				
Mean (sd)	18.7 (9.8)	16.5 (7.7)	15.8 (9.6)	12.2 (9.0)

Comparing research transfer activities by research domain and faculty we found several significant effects. As seen in Table 2, there was a significant main effect of research domain for Mode II activities, for both plain and engaged dissemination (both *p *< .001), with applied researchers reporting more of these activities than basic researchers. Main effects of faculty were limited to engaged dissemination (*p *= .025), with the medical school faculty demonstrating more of this dissemination than the other health science faculties. Moreover, faculty had a higher number of publications (*p *= .04), with the medical school faculty publishing more than other health science faculties. When considering Mode I activity, there was an interaction effect for research domain and faculty for number of scholarly publications (*p *= .01), in that applied researchers in medical faculties published more than their peers conducting applied research in other faculties.

**Table 2:** Comparison of health researchers by faculty and research domain using ANOVA^a^

	**Medical School**		**Other Faculty**		**Main Effects**		**Interaction**
	Applied	Basic	Applied	Basic	Domain	Faculty	Domain*Faculty

	Mean (Std)				F-statistic (p-value)		

**Dissemination Measures**							
Plain dissemination	14.2 (3.8)	9.7 (3.0)	15.4 (3.8)	9.8 (4.1)	88.9 (<.001)	3.4 (.07)	0.85 (.36)
Engaged dissemination	13.8 (4.7)	8.2 (3.1)	15.1 (4.5)	9.6 (4.7)	73.2 (<.001)	5.1 (.02)	0.01 (.93)
Number of publications	21.7 (13.8)	17.7 (11.3)	13.9 (12.0)	19.5 (13.0)	0.004 (.96)	8.35 (.004)	6.6 (.011)
**Other variables**							
Perceived importance of dissemination activities (Mode I)	11.8 (2.8)	10.8 (2.9)	12.02 (2.9)	11.0 (2.6)	6.80 (.01)	.36 (.55)	0.001 (.98)
Perceived importance of dissemination activities (Mode II)	11.8 (3.3)	10.1 (3.2)	11.4 (3.0)	9.3 (3.7)	15.9 (<.001)	1.9 (.17)	0.1 (.71)

^a^2-way ANOVA with Type II Analysis

As seen in Table 2, perceptions of the importance of translation activities were not statistically and significantly different for researchers when comparing by research domain and faculty. Rather, perceptions of the importance of Mode I and II activities were only significant according to the researcher's research domain. Applied researchers placed more importance on these activities than their basic researcher counterparts (Mode I, *p *= .01; Mode II, *p *< .001). No other main or interaction effects were significant in these analyses.

## Discussion

In this study, we examined health researcher characteristics and forms of research dissemination. With academic settings using traditional metrics (e.g., peer-reviewed publications, amount of grant dollars) for researcher evaluation, and major funding agencies developing key directives to facilitate knowledge translation with policy- and decision-makers, this exploration is timely. Our findings point to an emerging tension between the academic system versus the broader research funding context by illustrating important differences between health researchers, as well as providing insights into their professional characteristics and engagement in Mode I and Mode II activities.

While the differences among health researcher professional characteristics should be interpreted with caution, they do suggest future directions for research. The gender and academic rank differences between other applied research faculties and other faculties are notable when considering Mode I activity. In this sample, the faculty in which the researcher was located and their research focus was important: medical school researchers published more than researchers in other faculties (*p *= .004). Are researchers in other faculties engaging in fewer Mode I activities because of gender-related career disruptions (e.g., maternity leave), less protected time to conduct research and publish as a function of academic rank, or differing norms related to number of authors on a single publication? A future study collecting more specific data on gender and academic rank would answer these questions. Further, there may be differences in the ways in which medical school researchers are connected to health policy-makers and decision-makers that are not seen in other groups of researchers (e.g., membership to regional/provincial committees) that need to be identified as facilitators to Mode I and II activities.

When examining Mode activity and educational preparation together, there were several trends of interest. Mode I, or traditional knowledge production activity, was highest in the medical school applied research group – a group that reported more variation in their highest degrees obtained. Applied researchers in other health science faculties reported the least amount of scholarly publications with all graduate-level researchers. When examining whether the pattern of differences reported for publication output remained significant after accounting for experience, the pattern remained but the significance was not as strong, which indicates that years of experience explains only some of the observed differences in publication output. These trends suggest that publications may not be fully moderated by the level of educational training or years of experience, but may be influenced by experiences outside of the formal academic system. This may include post-training opportunities, such as research mentorship for junior researchers (e.g., their participation in more senior researchers' projects). There may be a role played by the researcher's faculty. For instance, in our sample medical school researchers may have been supported by an academic system following formal training that enabled them to publish more than other researchers. For researchers with low numbers of scholarly publications, the academy may need to consider peer relationships (i.e., mentoring, new research relationships) or infrastructure needed (i.e., protected time to write, supervisory relationships with graduate students) to promote publications.

In a comparison of Modes I and II activities by research domain and faculty, several significant effects were found. Research domain was linked to dissemination activities. As would be expected, compared to applied researchers, basic researchers reported significantly fewer Mode II activities (plain and engaged dissemination both *p *< .001). Given the access to a hospital setting, it is reasonable to suggest that a researcher holding positions in both academic and applied settings (university and hospital) would have greater success at directly translating research findings in a Mode II style than one who does not. However, this was not the case for our sample. Comparable proportions of basic and applied researchers worked in the university and hospital environments, suggesting an influence other than work environment for Mode II activity. The key may lie in building partnerships, and a more detailed examination of relational capital or working relationships may be useful. Given the time and commitment needed to engage in Mode II activities, the need for developing working relationships with research end-users to facilitate the application of research results has been emphasized [14,15]. The time spent on engaging in collaborative research transfer needs to be recognized as a valued researcher activity [9]. Under these circumstances, researchers would be supported in creating research environments that promote both Modes I and II activities. Debackere supports these types of organizational structure and management processes [16]. These relationships, however, should not be limited to end-users in the health services delivery or policy arenas, and should include relationships with other researchers, peers, industry partners, and administrative personnel who may provide linkages for engaging Mode II activity.

In this study, research domain mattered when it came to the importance placed on dissemination activity. Expectedly, applied researchers regarded more engaged activities, such as those related to Mode II knowledge production, as more important than basic researchers, and also engaged in more of this type of activity than their basic counterparts. These findings, however, may be more of a reflection of our survey question than the sample. We asked health researchers: "In terms of your professional satisfaction, what is the importance of workshops organized by users, participation in expert committees, etc.?" This survey question is worded in a way that lends itself more to applied researchers who study problems related to clinical care and the health care system, and see their dissemination audience as decision- and policy-makers who use workshops and expert committees as a means of addressing clinical practice and health systems issues. Basic researchers, on the other hand, may place importance on engaged dissemination activities more tailored to their field. For example, asking basic researchers: "In terms of your professional satisfaction, what is the importance of working with industry partners for licensing new products and patents, participation in biotechnology initiatives with industry, etc.?" may yield a different response than our study question. This expansion of how we defined engaged dissemination also may impact basic researchers' reporting of Mode II activities. A more in-depth understanding using a revised survey that is tailored to the basic and applied sciences, as well as qualitative research methods would permit further understanding of these researchers' activities, perceptions and values.

## Conclusion

In this paper, we examine the relationships between health researchers and researcher characteristics and forms of research transfer activities. Our findings point to two important areas for further exploration, research, and possible action.

First, in this study the importance of measurement is raised in terms of how 'engaged dissemination' (or Mode II activities) is defined to include the scope of both basic and applied researchers' activities. Methodological considerations are important, such as how best to measure Mode I and II production in a heterogeneous sample of health researchers. Use of a common measure would allow for the examination of sample (and sub-sample) differences, including potential covariates across more varied institutions, which can yield richer insights and enable an examination of relative influences, needs, and costs of each mode of activity.

Second, our findings illustrate important differences between and among health researchers and provide exploratory insights into what influences and characterizes researchers who engage in Modes I and II activities. If these differences observed in Alberta persist in other jurisdictions and times, it is important to understand further the contextual elements in health research, including relationships (social capital), organizational impact, and individual characteristics (i.e., academic training, years of experience). Understanding how these elements affect health research knowledge production is a necessary step in order to plan incentives and programs designed to influence dissemination activity. Clarifying factors that enable the system to nurture and support academy activity aligned with the current funding and policy environments is critical to ensuring return on resources invested in research.

## Abbreviations

CIHR, Canadian Institutes of Health Research; CHSRF, Canadian Health Services Research Foundation.

## Competing interests

The author(s) declare that they have no competing interests.

## Authors' contributions

CAE, PN and JMB participated in the design and conduct of the study. MSN led manuscript writing and statistical analytic design. AJA performed and assisted in interpreting the statistical analysis. RT participated in the conduct of the study. All authors read and approved the final manuscript.

## Supplementary Material

Additional File 1 Description of Variables. The table provided illustrates the survey variables, their items and the scaling used for data collection. Click here for file
