# Supplementary material for: Health researchers in Alberta: an exploratory comparison of defining characteristics and knowledge translation activities
Source: Implement Sci. 2007 Jan 4;2:1. doi: 10.1186/1748-5908-2-1 (PMC1781466; doi:10.1186/1748-5908-2-1)
Supplement: Additional File 1 — Description of Variables. The table provided illustrates the survey variables, their items and the scaling used for data collection. [file 1748-5908-2-1-S1.doc]

**Additional File 1: Description of variables**

| **Index** | **Item** | **Sub-items** | **Method (Range)** |
| --- | --- | --- | --- |
| ***Dependent Indices/Variables*** | | | |
| Plain dissemination | How frequently do you personally … | - Present research results in non-technical language to users - Provide examples or demonstrations of how to use research results - Disseminate reports and products appealing to target audience (graphics, color, humor, packaging) - Prepare reports on specific topics for policy makers - Send research results to physicians, health care professionals or managers of health delivery organizations (in the last five years) | (1=Never to 5=Very often)  Sum (5-25) |
| Engaged dissemination | How frequently does … | - Your project ensure personal follow-up with users to assess how useful project information is - Your project ensure personal follow-up with users to assess how easily project information could be applied - Your project involve users as co-investigators - Your project involve users in advisory committees - Your project involve users in definition of the research question | (1=Never to 5=Very often)  Sum (5-25) |
| Number of publications | What is the total number of your peer reviewed publications during the last five years? |  | “as is” |
| ***Independent Indices/Variables*** | | | |
| Perceived importance of dissemination activities (Mode I) | In terms of your professional satisfaction, what is the importance of … | - Original studies leading to scientific publications - Articles in trade journals - Putting project information on the Internet - Publication of articles in newspapers | (1=Not important at all to 5=Extremely important)  Sum (4-20) |
| Perceived importance of dissemination activities (Mode II) | In terms of your professional satisfaction, what is the importance of … | - Workshops organized by users - Participation in experts groups, expert committees - Production of newsletter and information delivered directly to intended users - Sending of reports to users by e-mail | (1=Not important at all to 5=Extremely important)  Sum (4-20) |
| Education | Most advance university degree completed? | - Bachelor - Master - PhD | “as is” |
| Academic rank | What is your academic rank? | - Full Professor - Associate Professor - Assistant Professor | “as is” |
| Years of experience | Number of years of experience in research after graduate researcher work |  | “as is” |
| Years of experience | Number of years of experience as service provider |  | “as is” |
| ***Derived Variables*** | | | |
| Research domain | Dichotomous variable:   - Applied research - coded ‘1’ - Basic research - coded ‘0’ | | |
| Faculty Domain | Dichotomous variable:   - Medical school - coded ‘1’ - Other faculties - coded ‘2’ | | |
| Work setting | ‘Tri-chotomous’ variable:   - Hospital only - coded ‘0’ - University only - coded ‘1’ - University + Hospital – coded ‘2’ | | |
